# Dull to Social Acceptance Rather than Sensitivity to Social Ostracism in Interpersonal Interaction for Depression: Behavioral and Electrophysiological Evidence from Cyberball Tasks

**DOI:** 10.3389/fnhum.2017.00162

**Published:** 2017-03-31

**Authors:** Qing Zhang, Xiaosi Li, Kai Wang, Xiaoqin Zhou, Yi Dong, Lei Zhang, Wen Xie, Jingjing Mu, Hongchen Li, Chunyan Zhu, Fengqiong Yu

**Affiliations:** ^1^Laboratory of Cognitive Neuropsychology, Department of Medical Psychology, Anhui Medical UniversityHefei, China; ^2^Anhui Mental Health CenterHefei, China; ^3^Department of Neurology, The First Affiliated Hospital of Anhui Medical UniversityHefei, China; ^4^Department of Psychiatry, The Chaohu Affiliated Hospital of Anhui Medical UniversityChaohu, China

**Keywords:** depression, event-related potential (ERP), social inclusion, social exclusion, cyberball

## Abstract

**Objectives:** Impairments in interpersonal relationships in depression present as irritability, pessimism, and withdrawal, and play an important role in the onset and maintenance of the disorder. However, we know little about the neurological causes of this impaired interpersonal function. This study used the event-related brain potential (ERP) version of the Cyberball paradigm to investigate the emotions and neural activities in depressive patients during social inclusion and exclusion simultaneously to explore neuropsychological mechanisms.

**Methods:** Electrophysiological data were recorded when 27 depressed patients and 23 healthy controls (HCs) performed a virtual ball tossing game (Cyberball) during which the participants believed they were playing with two other co-players over the internet. The Cyberball paradigm included two other conditions; inclusion during which participants received the ball with the same probability as the other players to experience a feeling of acceptance, and exclusion during which the participants experienced a feeling of ostracism when the other two players threw the ball with each other. The Positive and Negative Affect Schedule (PANAS) was used as a baseline and after each block during the Cyberball to assess positive and negative effects. In addition, a brief Need-Threat Scale (NTS) was used to assess the fulfillment of basic needs of subjects after each block and 10 min after ostracism. Moreover, the relationship between the ERP data of depression and clinical symptoms was analyzed.

**Results:** Exclusion compared to inclusion Cyberball caused a decrease in positive affect and an increase in negative affect. The group differences were only found in the positive affect. Moreover, patients reported a lower level of basic needs than did HCs after social inclusion, but a similar level of basic needs after social exclusion. At the electrophysiological level, patients showed decreased P3 amplitudes compared to HCs in social inclusion, and P3 amplitudes were borderline negatively correlated with their scores of anhedonia symptoms.

**Limitations:** A limitation of our study was that the subjects' criteria were different.

**Conclusions:** The behavioral and electrophysiological results indicated that the interpersonal problems in depressive patients were mainly due to deficits in processing the pleasurable social stimuli rather than aversive social cues.

## Introduction

Depression is a very common disease with high morbidity, disability, and recurrence rate (Luppa et al., 2007). It was the second biggest contributor to the disease burden in China between 1990 and 2010 (Yang et al., 2013). Part of the burden relates to the impairment in the quality of life and relationships for patients with depressive disorder (Mehta et al., 2014). The interpersonal relationships of depressed patients are presented as irritable, pessimistic, and withdrawn, which may be persistent or may recover more slowly than symptom changes (Southwick et al., 2005). These interpersonal problems that may be due to negative cognitive deviation, anhedonia, and emotional regulation deficits have important roles in the onset and maintenance of the depressive state. In addition, many effective short-term treatment strategies target the improvement of interpersonal problems, such as interpersonal psychotherapy (IPT) (Bruijniks et al., 2015). However, the neuropsychological mechanisms of this impaired interpersonal function in depression are poorly understood.

In daily life, ostracism, social exclusion, and rejection are common aversive phenomena during interpersonal interactions. Recently, studies have shown that social exclusion experiences are related to high accident rates, suicide, homicide, and increasing prevalence of affective disorder and personality disorder (Leary et al., 2003; Williams, 2007; Munjiza et al., 2014). Depressive patients may be sensitive to social exclusion. Jobst et al. found that social exclusion experiences elicited pronounced negative emotions and lower oxytocin levels in patients with chronic depression compared to healthy control subjects, suggesting that depression causes difficulty in coping adequately with aversive social cues (Jobst et al., 2015). Chronic exposure to neglect and rejection showed a strong association with the onset of depression (Slavich et al., 2010; Mandelli et al., 2015). Masten et al. conducted a neuroimaging study involving 20, 13-year-old adolescents who were included and excluded by peers during the Cyberball paradigm, and depressive symptoms were assessed via parental reports at the time of the scan and 1 year later. The results showed exclusion invoked greater sub anterior cingulate cortex (subACC) activity than inclusion, and that this activity was associated with increases in parent-reported depressive symptoms 1 year later (Masten et al., 2011). Thus, depression is closely interrelated with social exclusion.

In addition, it is noteworthy that depressive disorder was also found to be associated with impairments in generating positive emotions or motivations, which was named anhedonia (Watson and Clark, 1995; Sloan et al., 2001; Dichter et al., 2004; Joormann and Gotlib, 2006; Watson and Naragon-Gainey, 2010). Researchers conducted numerous studies using different paradigms to explore the anhedonia in depression and proposed that anhedonia in depression involved the impairment of motivation, reinforcement learning, and reward-based decision making, rather than the experience of pleasure *per se* (Pizzagalli, 2014). Studies showed that patients with depression had a response bias against happy expressions (Surguladze et al., 2004), and reported blunted affective responses to positive but not negative cues (Sloan et al., 2001; Rottenberg et al., 2002; Dichter et al., 2004). Moreover, studies suggested that reduced positive self-image, but not increased negative self-image predicted depressive symptoms 9 months later (Dobson and Shaw, 1987; Johnson et al., 2007). Using monetary reward tasks, Knutson et al. reported that patients showed significantly reduced reward responsiveness (Knutson et al., 2008). Another study reported that outpatient individuals who had high depressive symptoms showed lower reaction bias to high risk reward stimulation, which could predict their severity of future depression (Pizzagalli et al., 2005). Therefore, anhedonia was recognized as a key trait related to vulnerability of depression. Research on anhedonia represents a focus shift from the aspects of depression related to negative affect, to the aspects of depression related to positive affect (Forbes, 2009). Thus, relative to social exclusion, social inclusion, which means the perception of positive involvement in interpersonal interactions, is of even greater concern (Parr et al., 2004). However, previous research on anhedonia in depression mostly focused on pleasant images, words, or money as positive stimuli (Wang et al., 2006; Bylsma et al., 2008; Knutson et al., 2008; Foti and Hajcak, 2009). Few studies have addressed the processing of positive social stimuli which could be recognized as social reward stimuli, as critically important types of rewards in depression (Forbes, 2009). Therefore, studies on dysfunctional neural responses to social inclusion in depression are of great significance.

The aim of the present study was simultaneously focused on social ostracism and acceptance, using the Cyberball paradigm to address the behavioral and the neural activity during interpersonal interactions in patients with depressive disorders. The Cyberball paradigm is a computer-based virtual ball-tossing game (Williams et al., 2000). It is widely used for reliably inducing feelings of interpersonal ostracism and acceptance in the laboratory environment (Eisenberger et al., 2003; Sebastian et al., 2010; Bolling et al., 2011; Maurage et al., 2012; Mooren and van Minnen, 2014). In the ball-tossing game, there are two social situations, one is mental acceptance, and the other is mental ostracism. In mental acceptance, participants are included in the game and the rate of receiving the ball is the same as the other player. However, in mental ostracism, the player is excluded from the game and has no chance to receive the ball from the other player. To measure the immediate effects of the game, participants are asked to report how they felt regarding mood and four basic needs, including belonging, self-esteem, control, and meaningful existence (Jamieson et al., 2010). Furthermore, social exclusion induced an automatic emotion regulation process (DeWall et al., 2011). The four primary needs damaged during ostracism were recovered after a 45 min delay. This delay of effects of social exclusion represented the self-regulatory ability. Participants with high social anxiety reported a prolonged recovery compared to those with low social anxiety (Zadro et al., 2006). Patients with depressive disorders were dysfunctional in the automatic regulation of emotion (Kupfer et al., 2012). Therefore, we suggest that the reflexive and painful response to ostracism of patients with depression may be prolonged.

Event-related brain potentials (ERPs), known for their optimal temporal resolution on the millisecond scale, can provide information regarding the discriminative ability of the brain and neurocognitive processing related to shifting attention (Singh and Telles, 2015). ERPs can monitor the neural processes engaged in disrupted cognitive function and can identify specific neurocognitive deficiencies in mental patients (Campanella, 2013; Delle-Vigne et al., 2014). Therefore, it may be helpful for psychiatrists to better understand the pathophysiological mechanisms involved in diverse mental diseases and then develop a follow-up rehabilitation plan specific for each patient's deficit. Moreover, ERPs also can assess the possible benefits of training of the impaired cognitive functions by comparing them to absolute medicine therapy (Campanella and Maurage, 2016). Therefore, ERPs can be useful for clinicians to install a best suited individualized treatment. Thus, electrophysiological data is recorded when participants perform the Cyberball game to investigate the dynamic and ongoing neural processes associated with social interactions (Themanson et al., 2013, 2015). Prior reports reported that N2, P3b, and frontal slow wave in response to each exclusionary cue were closely related to the detection, appraisal, and regulation processes of social exclusion, respectively (Crowley et al., 2009; Themanson et al., 2013). Moreover, the P3b amplitude for the exclusionary cue, not the N2 component, was significantly correlated with a self-reported affect. Themanson therefore reported that the perception of being excluded may be more closely related to self-reported feelings in response to social exclusion (Themanson et al., 2013). Niedeggen reported that P3 in response to an inclusionary cue was related to the subjective expectancy of social involvement (Niedeggen et al., 2014). The current study should therefore observe the P3 component to investigate the neural basis of social involvement in depression.

Based on the above, we propose that: (i) patients with depression are hypersensitive to social exclusion during behavioral and neural activity; (ii) depression should show a blunt response to a social acceptance signal; and (iii) decreased need fulfillment induced by social exclusion should last longer in depressive patients compared to healthy control subjects.

## Methods

### Participants

Thirty-one outpatients with depression and 25 healthy control (HC) participants were included in the study. Four patients and two controls were excluded, because of transpiration artifacts in the ERPs or due to quitting the study prematurely. Thus, the final participants consisted of 27 depressive patients (18 female) and 23 HCs (17 female). The two groups were matched by age and years of education (see Table 1 for demographic characteristics). The patients were recruited at the Mental Health Center of Anhui Province of China and were diagnosed, by two senior psychiatrists, with depressive episodes without psychotic symptoms according to the DSM IV-TR. General exclusion criteria for participants included fewer than 6 years of education, younger than 18 years, older than 45 years, a history of organic brain disease or neurological disorders (e.g., dementia, epilepsy, or history of brain injury), or current or past substance abuse or dependence. Patients were also excluded if they suffered from any other psychiatric disorder except depression. The HCs were recruited from the website, and excluded if they had a current or past psychiatric disorder, or ever received psychiatric treatment. Within the depressive patients group, seven were medicated with a selective serotonin reuptake inhibitor (SSRI), four with a selective serotonin and noradrenaline reuptake inhibitor (SSNRI), one with tricyclic antidepressants (TCAs), and 12 patients were drug free. The study was approved by the local ethics board. All participants provided written informed consent. The healthy controls received 100 RMB for experiment compensation. Every participant completed a simple demographics questionnaire, including general information, psychiatric treatment history, and substance abuse or dependence history. The severity of current depressive symptoms was assessed with the Beck Depression Inventory 13 (BDI-13) which had satisfactory reliability and validity (Beck et al., 1961). The self-report measures consisted of 14 items based on the experience of the past 2 weeks, including the start day. The fourth item (life satisfactions), the eighth item (socializing), and the thirteenth item (appetite) were considered as anhedonia symptoms.

**Table 1:** **Demographic and clinical data**.

**Characteristics**	**Group; mean (*SD*)**		**Test statistic**	***P*-value**
	**D (*n* = 27)**	**HC (*n* = 23)**		
Age (years)	33.33 (8.086)	31.61 (6.528)	*t* = 0.82	0.416
Gender (*n*)	9 males	7 males	X^2^ = 0.048	0.827
Education (years)	13.67 (3.317)	12.91 (3.029)	*t* = 0.833	0.409
BDI	13.44 (6.30)	3.60 (3.20)	*t* = 6.771	<0.001

### Cyberball manipulation

**Figure 1:**
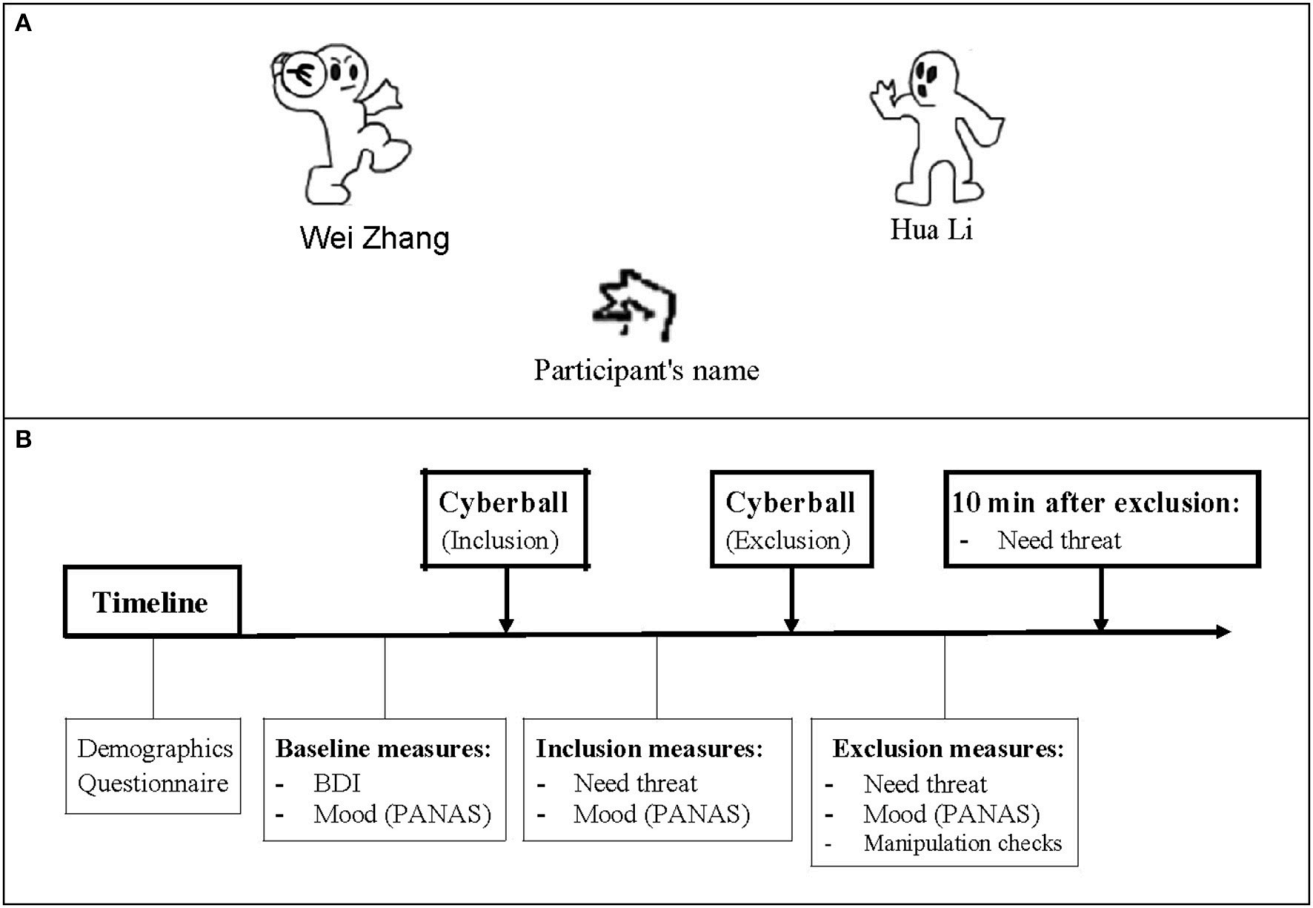
**The Cyberball game and a schematic of the experiment program**. **(A)** The Cyberball game: Participants are represented by a cartoon of hand at the bottom of the screen, and computer-generated players stand on either side. During the inclusion block, participant has a 50% chance of receiving the ball at each throw. While in exclusion condition, participant cannot catch the ball after receiving 10 throws. **(B)** A schematic of the time course of the experiment. After completing the demographics and basic measures, participant was asked to play the Cyberball game and report how they felt about the game. 10 min after the exclusion block, the Need-Threat Scale (NTS) was retested.

Participants were told that they would be playing an online game of “catch the ball” with two other players who connected over the internet, and that it did not matter who threw or caught, but rather that they used the animated ball toss game to assist them in visualizing the other players, the setting, and the temperature. Unknown to the participants, the two other players in the Cyberball game were computer-generated players controlled by a computer program. During the Cyberball game, the participant's neuroelectrical activity was recorded. The set of our Cyberball paradigm, including the number of throws, the time course, and the event-related markers, used the Themanson JR's ERP version of Cyberball (Themanson et al., 2013), but only two blocks (inclusion and exclusion) were administered (Figure 1). The detailed sets were that each block concluded after 80 trials, and the participant had a 50% chance of receiving the ball at each throw in the inclusion block, resulting in each participant getting ~33% of the throws. When the subject received the ball, he/she could press the F key if they wanted to throw the ball to the player on their left and the J key if right. Every trial lasted 2.5 s, including a 1.5 s period of ball movement, and 0.5 s before and after throwing the ball. For the two computerized players, random intervals between 0.5 and 3 s were set to create a sense that they were making a choice about throwing to a player. In the exclusion block, participants could not catch the ball after he/she received 10 throws, resulting in almost 50 exclusionary throws. The event markers were inserted at the time when the computerized players decided to throw the ball, and then the inclusion events were those throws from the computerized player to a participant during the inclusion block. The exclusion events were those between two computerized players during the exclusion blocks.

### Positive and negative affect scales

The Chinese version of the Positive and Negative Affect Schedule (PANAS) was also used. It was administered before the Cyberball task to assess the baseline status, and after social acceptance and exclusion tasks during the experiment.

### Assessment of basic needs

The brief Need-Threat Scale (NTS) used in the Cyberball paradigm has been previously reported (Williams et al., 2000; Zadro et al., 2004), and has been translated from English to Chinese and back-translated from Chinese to English until an agreement was found, including both the instructions and questionnaires. This back translation methodology was used by Brislin, and was a useful method to translate international questionnaires (Brislin, 1970). Participants were asked to complete the NTS after each Cyberball block according to how they felt while playing the game, whereas completing the NTS 10 min after the exclusion Cyberball block described how they felt “right now.”

### Manipulation checks

Manipulation checks were conducted to confirm the participant's exclusionary perception. Patients or participants were asked to estimate the percent of throws they had received and rate how much they felt excluded while playing the Cyberball game on a five point Likert Scale ranging from 1 (very included) to 5 (very excluded).

### Event-related potential recording

Electroencephalography (EEG) was recorded from 64 scalp sites using Ag-AgCl electrodes mounted on an elastic cap (Neuro Scan, Sterling, VA, USA) according to the international 10/20 system, with the left mastoid as reference and averaging of the left and right mastoids offline as a re-reference, as well as a forehead ground. Vertical and horizontal bipolar electrooculography (EOG) activity was recorded to monitor eye movements. EEG and EOG activity were continuously digitized (500 Hz sampling rate) and low-pass filtered (30 Hz; 24 dB/octave). All electrode impedances were maintained below 10 KΩ. Offline processing of the stimulus-locked ERP included ocular artifact removal using a regression procedure implemented in the Neuroscan software (Semlitsch et al., 1986), 1200 ms stimulus-locked epochs (a 200 ms pre-stimulus baseline), and artifact rejection (epochs with signals that exceeded ± 100 μV were excluded from averaging).

### Statistical analysis

Behavioral measures were statistically evaluated using SPSS, version 20 (IBM, Armonk NY, USA). Analysis of variance was performed using repeated measures analyses of variance (ANOVAs), two-tailed independent samples *t*-tests used Bonferroni correction, and Pearson's correlation analyses. An experiment-wise alpha level of *P* < 0.05 was set for all analyses. After visual inspection of the grand average wave forms (**Figure 4**), we detected a broad positive wave, which peaked around 420 ms after stimulus onset and clearly differentiated between the two groups and stimulus categories and electrodes. Therefore, we computed the average amplitude in the discrete latency window running from 370 to 470 ms after the event marker at electrode points of FZ, FCZ, and CZ.

## Results

### Behavioral measures

**Figure 2:**
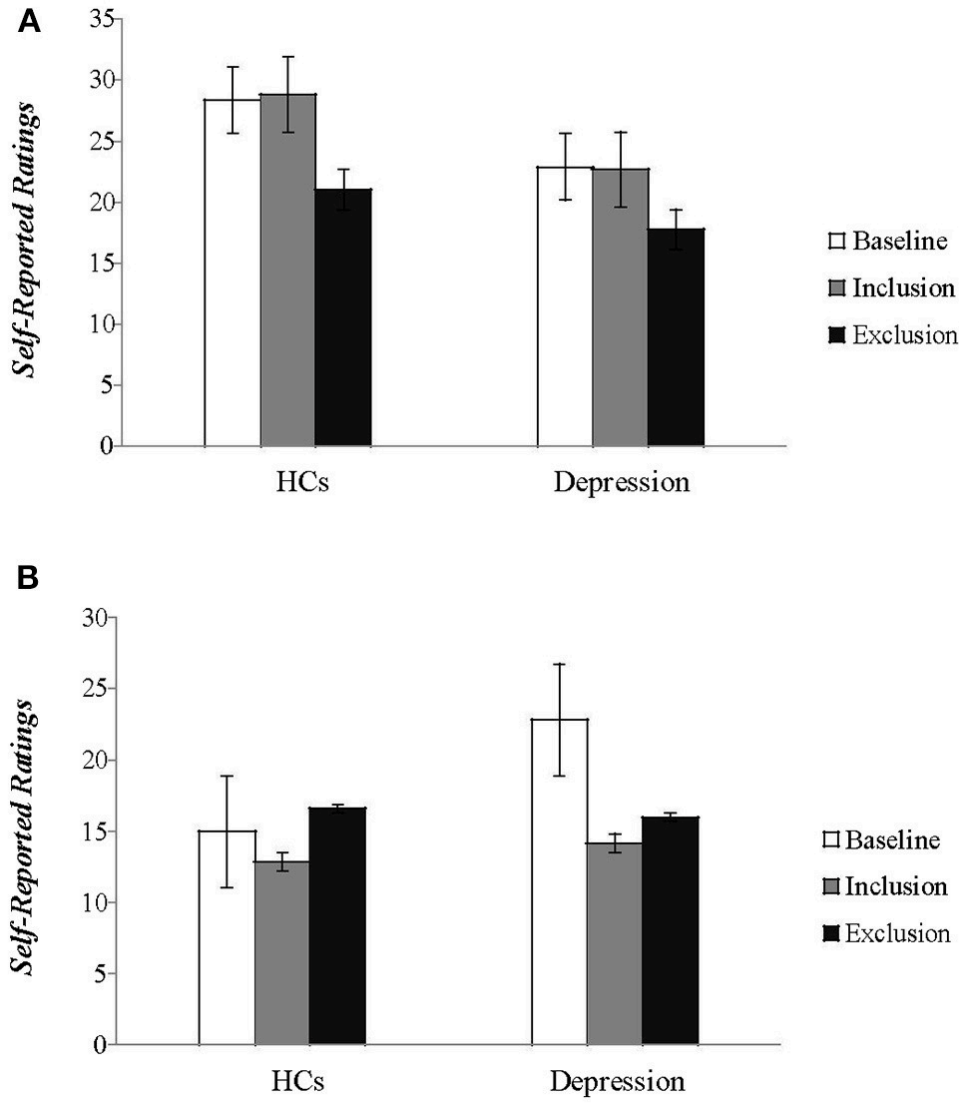
**The self-reported feelings of patients and healthy controls on the two subscales of the (A)** Positive and **(B)** Negative Affect Schedule (PANAS) during baseline and two blocks of the Cyberball tasks are plotted. (HCs, healthy controls).

Statistical analyses on scores of PANAS showed the expected blocking effects on both positive affect (PA) and negative affect (NA) [*F*_(1, 49)_ = 67.437, *P* < 0.001; *F*_(1, 49)_ = 18.020, *P* < 0.001], and a significant group effect on PA [*F*_(1, 49)_ = 7.357, *P* < 0.01]. But the group effect on NA [*F*_(1, 49)_ = 0.072, *P* > 0.05] and the block group interaction effect [*F*_(1, 49)_ = 3.407, *P* > 0.05; *F*_(1, 49)_ = 2.163, *P* > 0.05] were not significant. At baseline, patients scored lower than HCs on positive affect [*t*_(49)_ = −2.928, *P* < 0.01] and higher on negative affect [*t*_(49)_ = 4.090, *P* < 0.01] (Figure 2). These findings suggest that social exclusion resulted in a significant decrease in positive affect and increase in negative affect for both patients and HCs. The positive moods of patients were lower than the HCs after measurements following social inclusion and exclusion, respectively; the negative mood of the patients showed no significant difference from the HCs.

**Figure 3:**
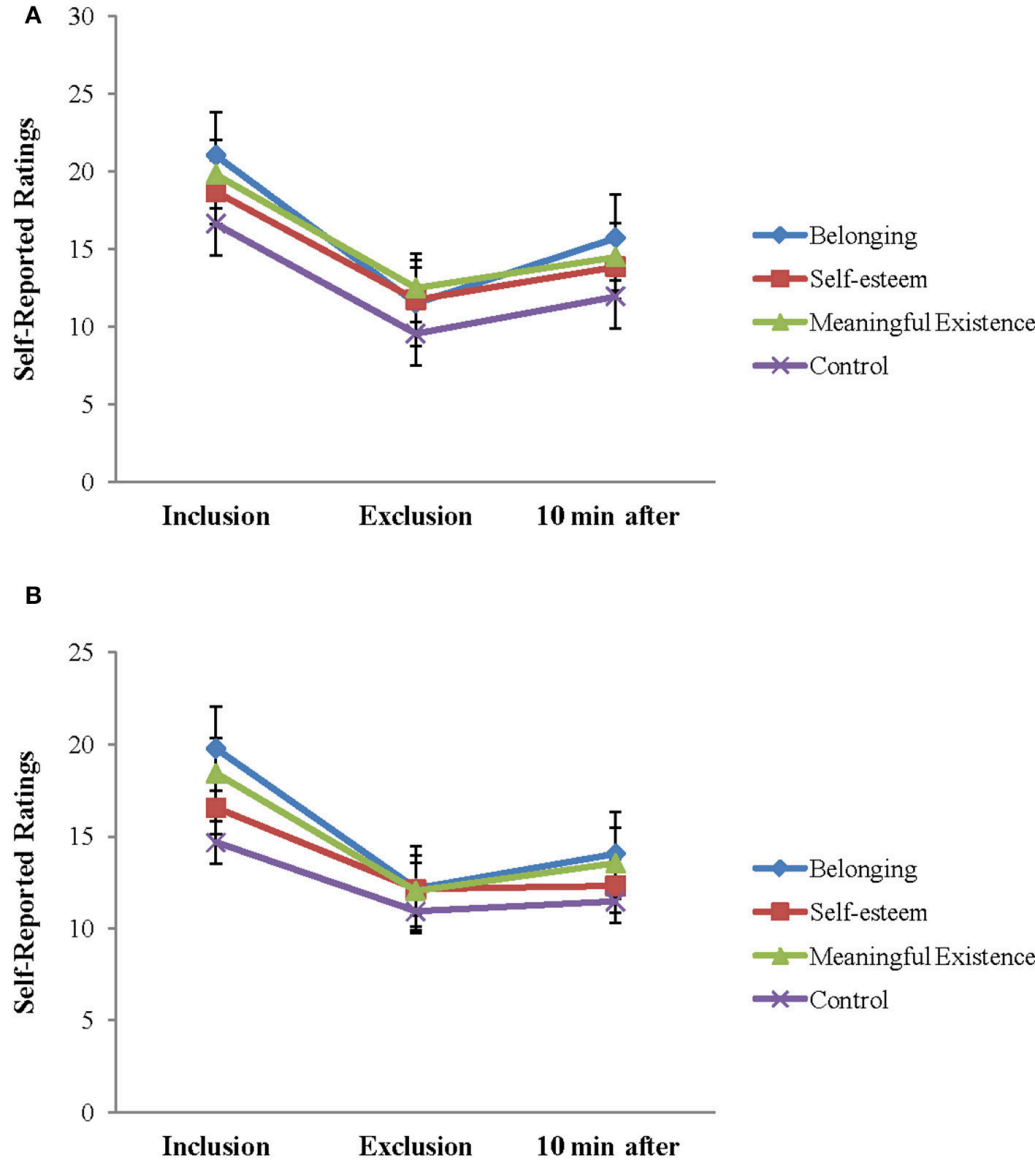
**Self-reported feelings on the Need-Threat Scale after each Cyberball block and 10 min after exclusion Cyberball block are shown for each group separately (HCs, healthy controls). (A)** NTS in HCs and **(B)** NTS in depression.

The repeated ANOVA of 3 (block: Inclusion, exclusion, and 10 min after exclusion) × 2 (group: Patients and HCs) analysis on scales of NTS also revealed the expected block effects for all the four fundamental human needs (belonging, self-esteem, control, and meaningful existence): *F*_(2, 48)_ = 111.659, *P* < 0.001; *F*_(2, 48)_ = 70.214, *P* < 0.001; *F*_(2, 48)_ = 57.978, *P* < 0.001; *F*_(2, 48)_ = 61.034, *P* < 0.001, and block group interaction effects for self-esteem and control needs were also significant [*F*_(2, 48)_ = 3.407, *P* < 0.05; *F*_(2, 48)_ = 5.446, *P* < 0.05]. The group main effects for the four basic needs were not significant (*P*-values > 0.1). Follow-up analyses showed that patients were more threatened on self-esteem and control needs during social inclusion [*t*_(49)_ = –2.193, *P* < 0.05; *t*_(49)_ = −1.916, *P* = 0.061], but were not different from HCs during social exclusion. Further simple analyses showed a significant decrease of scores on self-esteem and control needs between the inclusion and exclusion block and an increase between the exclusion block and 10 min after exclusion for HCs (*P*-values < 0.01). For patients, there were no significant differences for self-esteem and control needs between the exclusion block and 10 min after exclusion (*P*-values > 0.1) (Figure 3). The findings suggested social exclusion could induce a significant decrease in all needs fulfillment for both groups. After 10 min, these effects were restored in HCs, but not in patients with depression, especially on the subscales of the self-esteem and control needing parameters.

### Neural measurements

**Figure 4:**
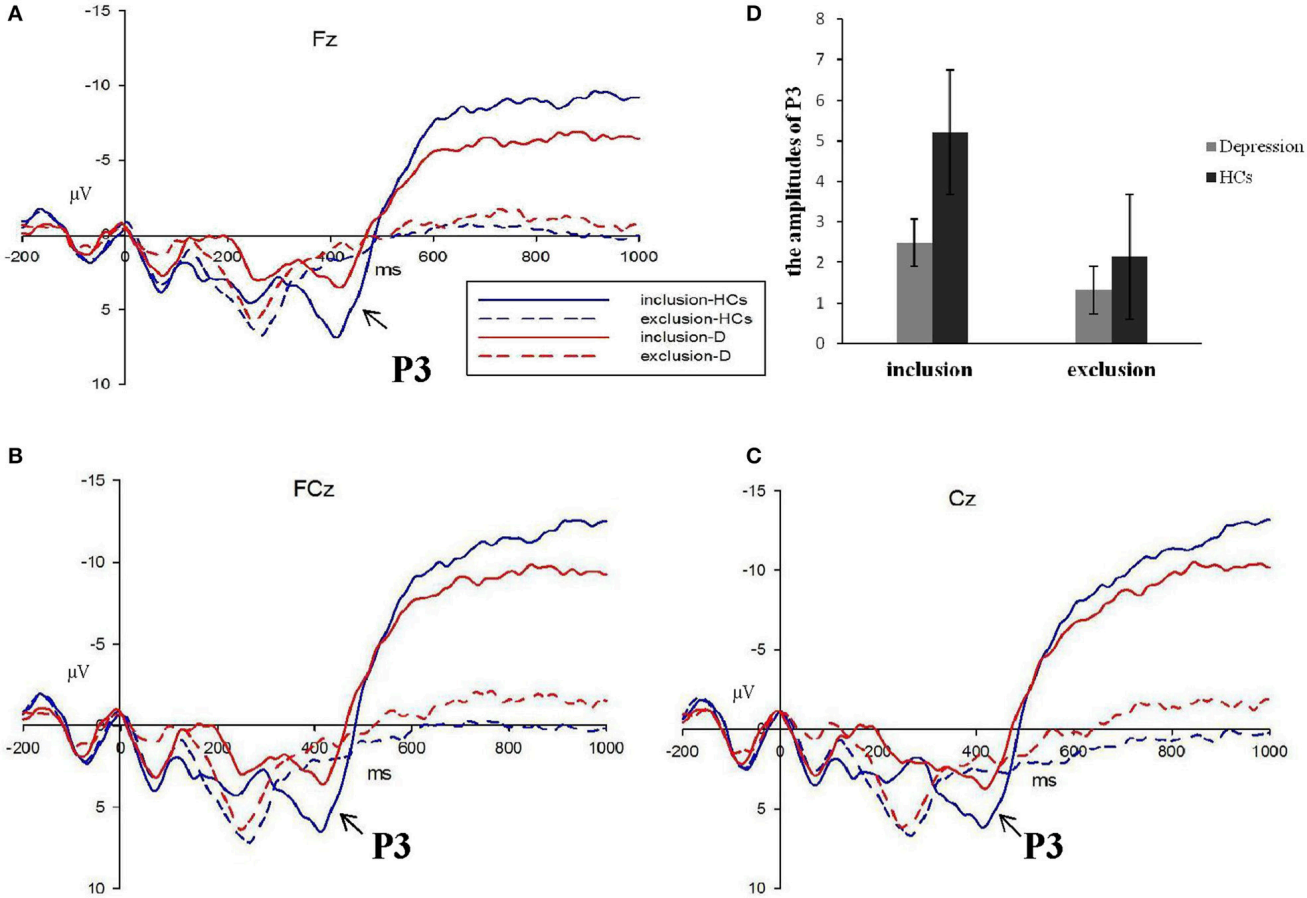
**The P3 average waves evoke d by inclusion and exclusion in depressive patients (red lines) and healthy controls (blue lines) at Fz, FCz, and Cz sites (A–C)**. The corresponding amplitudes histogram was shown atthe upper right **(D)**. (HCs, healthy controls; D, depressive patients).

**Figure 5:**
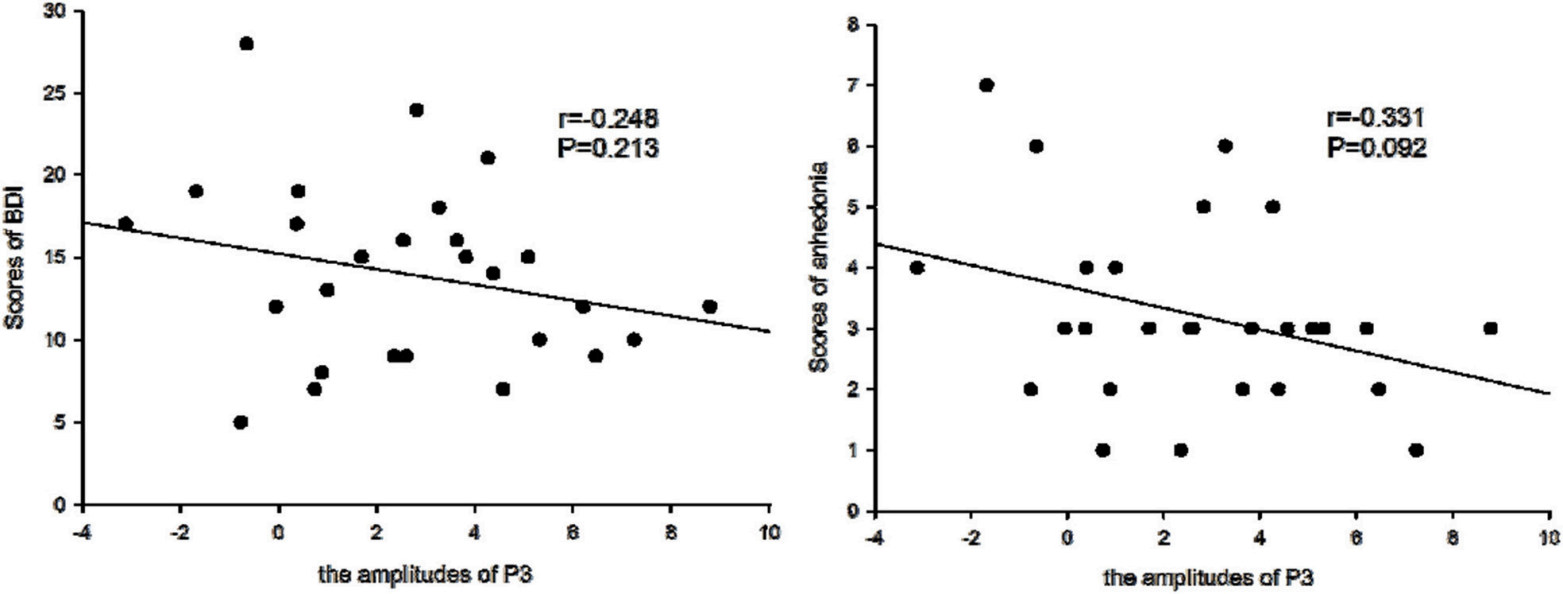
**Amplitudes of P3 and BDI symptoms**. The amplitudes of P3 were not correlated with the scores of Beck Depression Inventory (BDI), but were borderline negatively correlated with the scores of anhedonia symptoms for patients with depression.

Average wave forms elicited by two stimuli at FZ, FCZ, and CZ are shown in Figure 4. Omnibus 2 (block: Inclusion and exclusion) × 2 (group: Patients and HCs) × 3 (electrode: FZ, FCZ, and CZ) repeated measures analyses of variance (ANOVAs) were conducted to compare the average amplitude of P3 components (wave-forms between 370 and 470 ms) across different blocks, electrodes, and groups. Results showed a significant effect for block [*F*_(1, 49)_ = 6.794, *P* < 0.05], electrode [*F*_(1, 49)_ = 5.363, *P* < 0.01], and group [*F*_(1, 49)_ = 5.646, *P* < 0.05], but the interaction effect of the block group was not significant (*F*_(1, 49)_ = 1.360, *P* > 0.05). Considering that the group and block interaction effects on PA and NTS scores were significant, we analyzed the group effect of social inclusion and social exclusion on P3, respectively. Results showed a less positive-going wave for patients during inclusion Cyberball [*F*_(1, 49)_ = 4.269, *P* < 0.05]. The differences on P3 for exclusion Cyberball categories were not significant [*F*_(1, 49)_ = 0.994, *P* > 0.05] (Figure 5).

### Correlation analysis

We determined that the average amplitudes of P3 evoked by inclusion were not significant and negatively correlated with BDI scales (*r* = –0.234, *P* > 0.05), but were borderline significant and negatively correlated with the scores of anhedonia items (*r* = –0.331, *P* = 0.092), which suggested that with higher scores of anhedonia items, P3 amplitudes would be lower.

## Discussion

The main findings of this study were that a significant difference exists both for subjective reports and electrophysiological activity in encoding pleasurable social stimulus rather than aversive social cues between depressive patients and HCs. Exclusion Cyberball compared to inclusion Cyberball caused a decrease in positive affect and an increase in negative affect and also decreased basic need satisfaction for both groups. When examining the group effect, the scores of negative subscales of PANAS did not differ between the two groups. However, the scores of positive subscales were both lower after playing inclusion and exclusion Cyberball in patients relative to HCs. In addition, the self-esteem and control needs in patients were lower in patients than for HCs after social acceptance, but not after social ostracism. More importantly, in electrophysiological levels, patients showed decreased P3 amplitudes compared to HCs in social acceptance conditions rather than in social exclusion conditions. P3 amplitudes evoked by acceptance were borderline negatively correlated with anhedonia scores in the patient group. A mechanism for understanding the impairment of social function of depression is discussed below.

This study found that the scores of patients with depression, for negative affect and primary needs measured after social ostracism block, were the same as those of HCs, suggesting that patients on the behavioral level were not more sensitive to social ostracism than HCs. There was also no significant difference in the P3 component response to social exclusionary events between patients and HCs. Because the detailed settings of the social exclusion block of our Cyberball paradigm were imitations of the Themanson JR's ERP version Cyberball, the P3 component, activated by exclusionary events, mainly represented the explicit awareness or perception of being excluded and the related allocation of attention to the exclusionary experience (Themanson et al., 2013). This result was inconsistent with our first hypothesis. The heterogeneity of patients may be one confounding factor affecting these results. More importantly, the relationship between social ostracism and depression could involve a model in which social ostracism events activated brain regions involved in negative effects, eliciting negative self-cognition, and released proinflammatory cytokines, with increased risk of depression (Slavich et al., 2010). We therefore suggest that the sensitivity to social ostracism depended more on stressful life events, which was consistent with a previous report (Iffland et al., 2014).

The main behavioral group effect of this study was found primarily in social acceptance, where patients scored lower on positive affect and basic needs compared to HCs. This indicated that patients with depression showed a dull response to positive cues. Our finding was consistent with a previous study (Sloan et al., 2001). This study reported that depressed women showed a reduced frequency and intensity only to pleasant stimuli, and the recall for only pleasant words was different with non-depressed women. More importantly, the electrophysiological results indicated that patients showed decreased P3 amplitudes in response to social acceptance, when compared to HCs. Studies on inclusion and overinclusion demonstrated overinclusion increased the satisfaction of primary needs and indicated that P3 amplitude was a signal of modulation of the subjective expectancy of involvement (Niedeggen et al., 2014). Our results therefore indicated that depressive patients expressed lower expectancy on social involvement during social acceptance.

It was further noted that the P3 amplitudes evoked by inclusion were borderline negatively correlated with the severity of anhedonia symptoms. Because anhedonia was recognized as the typical clinical symptom of depression, the P3 amplitude could be a prognostic index of anhedonia symptoms in depression. Furthermore, the electrophysiological method may be a more sensitive technique for predicting the prognosis of the lesion compared to the behavioral results, which did not correlate with depressive symptoms.

Our studies also found that exclusion led to detrimental effects on mood and the four measured human needs (belonging, control, self-esteem, and meaningful existence) in both patient and HCs groups, which were in agreement with previous studies (Zadro et al., 2006; Williams, 2007; Jamieson et al., 2010; Onoda et al., 2010; Domsalla et al., 2014). This indicated that social exclusion, which has been conceptualized as a significant threat to survival (Baumeister and Leary, 1995; Macdonald and Leary, 2005), could have a negative impact on psychological processes. Moreover, it is worth noting that in this study, patients with depression showed more prolonged negative effects of ostracism than did HCs after the social exclusion task. This persistence of the effect of exclusion, which was hypothesized to be a symbol of emotional dysregulation (Kashdan et al., 2006), was also found in individuals experiencing psychological difficulties, such as social anxiety and schizophrenia (Zadro et al., 2006; Perry et al., 2011). This can be explained by the fact that individuals with psychological vulnerability are more likely to obsess about negative social encounters. Davidson's view on plasticity in the neural circuitry of emotion could also provide an explanation. Mental disorders, especially mood disorders, are featured as expressing normal emotion in inappropriate contexts (e.g., expression of the negative effect of social ostracism in the context when mood has been appeased) (Davidson et al., 2000). The current study further suggested that future research on ostracism in patients with mental disorders requires assessing the effects of ostracism across time, rather than only focusing on immediate reactions.

This study had theoretical and clinical value for the involvement of anhedonia in depression. The results showed that depressive patients have deficits in processing pleasure rather than aversive social cues, indicating that anhedonia in depression could also be demonstrated in processing social reward stimuli. This supported the possibility that anhedonia is one of the most promising diagnostic endophenotypes of depression (Pizzagalli, 2014). Moreover, most of the previous studies measured anhedonia usually using face perception or emotional words as emotion-induced stimuli (Surguladze et al., 2004; Joormann and Gotlib, 2006; Wang et al., 2006). The present study further tested the anhedonia theory in the social interaction environment, which is more critical to human functioning. On a clinical level, psychotherapy on interpersonal dysfunction has been suggested to involve much more attention on the patient's lower expectance of involvement to social acceptance, to encourage patients to engage in rewarding social activities to moderate depression.

There are some limitations to our study. One is that the subjects' criteria were different. The subjects in the present study consisted of some chronic depression patients most of whom used medications, and some drug-free first episode patients. Thus, the drugs could have influenced anhedonia in social interactions. Another limitation is the poor spatial resolution of ERPs, which could not accurately discriminate activation of brain areas during social interactions. Future studies using other brain imaging methods, such as functional magnetic resonance imaging with high spatial resolution, are needed to substantiate and extend our findings.

## Conclusions

The present results demonstrated that when considering social acceptance and ostracism conditions simultaneously, patients with depression experienced lower positive effects and basic needs than did healthy control subjects mainly during social acceptance. The P3 amplitudes were significantly smaller in patients than in controls during social inclusion. In addition, the P3 amplitudes in patients, evoked by inclusion, were borderline negatively correlated with the severity of anhedonia symptoms. These findings indicate that the interpersonal dysfunctions in depressive patients are mainly due to anhedonia to socially rewarding stimuli rather than being sensitive to social rejection. The current study also provides behavioral and electrophysiological evidence that anhedonia is the endophenotype of depression.

## Ethics statement

The ethics board of Anhui Medical University. All subjects voluntarily joined this study with informed consents. Minors, persons with disabilities and endangered animal species were not involved in this study.

## Author contributions

CZ designed the study and wrote the protocol. XL, YD, XZ, WX, and JM undertook the patients recruiting. QZ and HL managed the literature searches and analyses. FY and LZ provided help for the data analysis. QZ wrote the first draft of the manuscript. KW and XZ assisted with the proof-reading of the manuscript.

### Conflict of interest statement

The authors declare that the research was conducted in the absence of any commercial or financial relationships that could be construed as a potential conflict of interest.
